# 恩诺沙星粉中未知添加物的确证

**DOI:** 10.3724/SP.J.1123.2020.09007

**Published:** 2021-06-08

**Authors:** Yue XIONG, Cheng WANG, Jianhui LIU, Huihui SHI, Yunhua WANG, Yao SUN, Jie YU

**Affiliations:** 1.江苏省兽药饲料质量检验所, 江苏 南京 210036; 1. Jiangsu Testing Institute of Veterinary Drug and Feed, Nanjing 210036, China; 2.上海爱博才思分析仪器贸易有限公司, 上海 200335; 2. Shanghai AB SCIEX Analytical Instrument Trading Co., Ltd., Shanghai 200335, China

**Keywords:** 超高效液相色谱, 二极管阵列检测, 飞行时间高分辨质谱, 亚硫酸氢钠甲萘醌, 恩诺沙星粉(水产用), 非法添加, ultra-performance liquid chromatography (UPLC), photo diode array detection (PAD), time of flight high resolution mass spectrometry (TOF-HRMS), menadione sodium bisulfite, enrofloxacin powder (used for aquaculture), illegal addition

## Abstract

应用非靶向分析技术,筛查、分析和确证恩诺沙星粉(水产用)中的非法添加物。分别制备甲酸酸化、碳酸钠碱化的恩诺沙星粉供试品溶液,经超高效液相色谱-二极管阵列检测器(UPLC-PDA)检测初筛,获取未知物色谱图。应用超高效液相色谱-飞行时间高分辨质谱(UPLC-TOF-HRMS),在正、负离子模式下对酸化、碱化样液进一步检测,获得未知物母离子和二级特征碎片的精确质荷比、同位素信息,并应用SCIEX OS软件进行分析推导。最后取疑似化合物对照品进行确证研究。UPLC-PDA初筛结果显示:酸化样液在1.870 min、5.122 min,碱化样液在5.122 min,均发现高响应未知色谱峰。对吸收波长和峰面积进行分析,推测含2个未知物,且未知物1(1.870 min)与未知物2(5.122 min)在酸性/碱性条件下可能发生转换。SCIEX OS软件分析推导结果显示:未知物2,母离子分子式拟合为C_11_H_8_O_2_,二级碎片结构解析含1个苯环、2个羰基和1个通过成环连接的丙烯结构,推测为甲萘醌;未知物1的分子离子峰为C_11_H_9_O_5_S^-^,二级碎片仅采集到HSO_3_^-^,丢失部分与未知物2一致,结合甲萘醌常见衍生物类型,推测为亚硫酸氢钠甲萘醌。取甲萘醌、亚硫酸氢钠甲萘醌对照品进行对比研究,UPLC-PDA检测结果显示:未知物1与亚硫酸氢钠甲萘醌、未知物2与甲萘醌,保留时间和紫外光谱一致;向供试溶液中添加对照品溶液后检测,未知物为单一峰。UPLC-TOF-HRMS检测发现:未知物1与亚硫酸氢钠甲萘醌保留时间一致,一级质谱质量偏差为1.0×10^-6^,二级谱库匹配度为100%;未知物2与甲萘醌保留时间一致,一级质谱质量偏差为0.6×10^-6^,二级谱库匹配度为99.7%。未知物1和2的结构得以确证。亚硫酸氢钠甲萘醌可止血,与恩诺沙星治疗败血症的适应证相应,佐证试验结果。随着对兽药非法添加行为的严格监管和严厉打击,非法添加物和添加手段愈发隐蔽,常规靶向分析难以满足监控需求。该文详述的使用UPLC-PDA结合UPLC-TOF-HRMS对未知物进行非靶向分析的技术,可为药品、食品、保健品、化妆品及农药等产品中非法添加物的筛查和确证提供思路和技术参考。

## Abstract (en)

In order to ensure the safety of animal food and regulate the application of veterinary drugs, it is necessary to strictly monitor their content, and to constantly improve the methods used to detect non-specific, illegally added substances in veterinary drugs. A study about the screening, analysis, and confirmation of illegal additives in enrofloxacin powder (used for aquaculture) using non-targeted analysis technology was introduced. First, an enrofloxacin powder test solution under acidic conditions was prepared by adding formic acid, and an enrofloxacin powder test solution under alkaline conditions was prepared by adding sodium carbonate. An ultra-performance liquid chromatography with photodiode array detector (UPLC-PDA) was used to assay the test solutions for the presence of unknown additives. Results revealed two high-response unknown peaks in the acidified test solution, with retention times of 1.870 min and 5.122 min respectively. In the alkalized test solution, only one high-response unknown peak was found, with a retention time of 5.122 min. The ultraviolet spectrum characteristic peaks at 5.122 min in acidified and alkalized test solutions were similar, but the peak area in the alkalized test solution was almost ten times that in the acidified solution. Two potential unknown substances were detected. Unknown substance 1 (1.870 min) and unknown substance 2 (5.122 min) may transform under acidic or alkaline conditions. Ultra-performance liquid chromatography-time of flight high resolution mass spectrometry (UPLC-TOF-HRMS) was used to analyze the unknown compounds in more detail. The acidified and alkalized test solutions were detected in the positive and negative ion modes of mass spectrometry, respectively. Accurate mass of the precursor ion, characteristics of secondary ion fragments, and isotopic intensity ratio of the two unknown substances were collected. This information was imported into SCIEX OS software. The molecular formula of the parent ion of unknown substance 2 was found to fit to C_11_H_8_O_2_, and its secondary fragment structure may contain a benzene ring and two carbonyl groups, with a propylene structure connected to them through ring formation. From this, unknown substance 2 was presumed to be a menadione. The molecular ion peak of unknown substance 1 was found to fit to C_11_H_9_O_5_S^-^, only HSO_3_^-^ was collected in the secondary fragments, and the missing part was consistent with unknown substance 2. Considering the most common derivatives of menadione, unknown substance 1 can be proposed to be menadione sodium bisulfite. Finally, we used menadione and menadione sodium bisulfite as reference substances in a comparative study. The same treatment method was used to prepare menadione, menadione sodium bisulfite reference solution, and enrofloxacin powder test solution. After UPLC-PDA detection, unknown substance 1 and menadione sodium bisulfite, unknown substance 2 and menadione, were found to have similar retention times and UV spectra. When the reference solution was added to the enrofloxacin powder test solution, the peak purity of the unknown substance did not change, and were all single peaks. UPLC-TOF-HRMS analysis revealed that the retention time of unknown substance 1 was consistent with that of sodium menadione bisulfite: compared to its accurate mass number in theory, the mass accuracy error was 1.0×10^-6^, and the matching degree of fragmentation information in the library was 100%. The retention time of unknown substance 2 was same as the menadione: compared to its accurate mass number in theory, the mass accuracy error was 0.6×10^-6^, and the matching degree of fragmentation information in the library was 99.7%. The structures of unknown substances 1 and 2 were confirmed.

Menadione sodium bisulfite is known to participate in the synthesis of thrombin in the liver, and also promotes the formation of prothrombin, and accelerates coagulation. The indication of enrofloxacin powder (used for aquaculture) is the treatment of hemorrhage and sepsis in aquaculture animals such as fish and eel. The pharmacological effects of the two drugs correspond to each other, which can cause producers to take risks and add them illegally. With the strict supervision and severe restrictions on the addition of veterinary drugs, illegal additives are becoming more and more subtle. Conventional targeted analysis does not always meet the monitoring requirements. In this paper, the non-targeted analysis of unknown substances using UPLC-PDA combined with UPLC-TOF-HRMS is described in detail. The results may provide a technical reference for screening and identifying illegal additives in drugs, food, health care products, cosmetics, and pesticides.

随着养殖业的快速发展,动物疫病的形势日趋严峻。为满足部分养殖者片面追求疗效、增加养殖密度等需求,少数兽药企业不按标准规定处方生产,随意改变产品配方或是非法添加其他药物,以期扩大产品销路,寻求更高收益。近年来,农业农村部已发布了50多种兽药非法添加物检查方法,但检测标准的制定速度仍然跟不上企业非法添加物调整变化的步伐^[1]^。2020年农业农村部公告第289号《兽药中非特定非法添加物质检查方法》^[2]^的颁布实施,为兽药中未知添加物的筛查与确证工作提供了判定依据,解决了目前兽药检测机构对于使用非标方法筛查出来的结果无法出具带有法律效应的报告,而使不合格兽药难以得到有效查处的尴尬局面。

目前,药品、食品、保健品、化妆品、农药中非法添加物的筛查确证主要基于两种模式。一是靶向筛查模式,利用高效液相色谱-二极管阵列检测(HPLC-PDA)法^[3,4]^、超高效液相色谱-质谱(UPLC-MS)法^[5,6]^、气相色谱-质谱(GC-MS)法^[7,8]^等收集化合物的保留时间、光谱图、母离子精确质荷比、二级碎片谱图等信息,建立数据库。再使用软件将样品数据与谱库进行比对,如有匹配结果,可在相同条件下进适宜浓度的对照品溶液获得相关谱图进行确证。靶向筛查法又可称为谱库比对法,未知物的检出率主要依赖数据库的大小,对于未收入谱库的化合物有漏筛的风险,存在一定的局限性。二是非靶向筛查模式,利用多种方法联用技术对谱库中未收集的化合物进行推测分析,常收集高分辨质谱提供的精确质荷比、同位素分布及二级碎片信息,使用软件拟合出未知物的分子式,并对主要二级碎片进行解析,推测未知物的主要官能团和裂解途径,推导出未知物的疑似结构,并设法获取相应对照品进行确证。有需要时,可通过制备液相色谱等手段对样品进行分离纯化,得到目标物后再利用紫外光谱、红外光谱、核磁共振谱、质谱等现代波谱解析方法进行结构预测,提高结果的准确度^[9,10]^。对于这种非靶向筛查,除了分析实验数据外,还要关注产品本身的疗效用途,结合商品名、包装宣传等信息,进行大胆合理的推断。

近日,在对一批兽药恩诺沙星粉(水产用)进行处方外非法添加物筛查时,发现高响应未知色谱峰。参考未知物非靶向筛查模式,通过分析高分辨质谱采集的数据,结合该产品的临床作用等信息,推导出疑似添加物,并用超高效液相色谱-二极管阵列检测器(UPLC-PDA)法和超高效液相色谱-飞行时间高分辨质谱(UPLC-TOF-HRMS)法进行双重验证,最终确证该批恩诺沙星粉中的非法添加物为亚硫酸氢钠甲萘醌。本文的研究具有较好的实践指导意义。

## 1 实验部分

### 1.1 仪器、试剂与材料

ACQUITY UPLC超高效液相色谱仪配PDA检测器及Empower 3 工作站(美国Waters公司); Triple TOF 4600超高效液相色谱-飞行时间高分辨质谱仪配OS及MasterView软件(美国SCIEX公司); XS205DU分析天平(瑞士Mettler Toledo公司)。甲醇、乙腈、甲酸为色谱纯(美国Merck公司);水为超纯水;其他试剂均为分析纯(南京化学试剂股份有限公司)。甲萘醌对照品购自中国兽医药品监察所(批号V0011304;含量99.3%);亚硫酸氢钠甲萘醌原料购自云南陆良和平科技有限公司(批号CVS20190906C;以C_11_H_9_NaO_5_S·3H_2_O计,为96.3%,作为对照品使用)。待测样恩诺沙星粉(水产用)为2019年度国家兽药风险监测样品(批号:1904011;规格:5%)。

### 1.2 实验条件

1.2.1 UPLC-PDA条件

ACQUITY UPLC BEH C18色谱柱(50 mm×2.1 mm, 1.7 μm);柱温:30 ℃;进样体积:2 μL;流速:0.3 mL/min;流动相A为0.1%甲酸水溶液,流动相B为0.1%甲酸乙腈溶液,梯度洗脱:0~7.0 min, 95%A~40%A; 7.0~7.5 min, 40%A~95%A; 7.5~10 min, 95%A。二极管阵列检测器,采集波长范围210~400 nm,分辨率1.2 nm,记录260 nm波长处的色谱图。用于未知物的初筛及最后的未知物确证。

1.2.2 UPLC-TOF-HRMS条件

ACQUITY UPLC BEH C18色谱柱(50 mm×2.1 mm, 1.7 μm);柱温:30 ℃;进样体积:10 μL;流速:0.3 mL/min;流动相A为0.1%甲酸水溶液,流动相B为0.1%甲酸乙腈溶液,梯度洗脱:0~12.0 min,95%A~75%A;12.0~16.0 min,75%A~45%A;16.0~18.0 min,45%A;18.1~20.0 min,95%A;二极管阵列检测器,记录260 nm波长处的色谱图(用于未知物结构分析时确定未知物在质谱图中的保留时间)。采用ESI离子源,分别在正、负离子模式下采集数据。正离子模式:电喷雾电压为5500 V,温度为550 ℃,雾化气体为N_2_,雾化气压力344.75 kPa(50 psi),辅助雾化气压力344.75 kPa(50 psi),气帘气为241.32 kPa(35 psi);一级质谱扫描范围为100~1000 Da,去簇电压为80 V;碰撞能量为10 eV;二级质谱扫描范围为50~1000 Da,去簇电压为80 V,碰撞能量为35 eV,碰撞电压扩展能量为15 eV;开启动态背景扣除(DBS)。负离子模式:喷雾电压为-4500 V;一级质谱去簇电压为-80 V,碰撞能量为-10 eV;二级质谱去簇电压为-80 V,碰撞能量为-35 eV;其他条件同正离子模式。

### 1.3 溶液的制备

1.3.1 酸性供试品溶液的制备

UPLC-PDA酸性初筛溶液: 称取供试品0.5 g,置具塞锥形瓶中,加甲酸-甲醇-水(1∶250∶250, v/v/v)溶液250.0 mL,摇匀,超声10 min,冷却至室温,滤过,取滤液,即得。

UPLC-TOF-HRMS酸性推导溶液: 用甲醇-水(1∶1, v/v)溶液将酸性初筛溶液稀释制成每1 mL约含恩诺沙星500 ng的溶液,即得。

1.3.2 碱性供试品溶液的制备

UPLC-PDA碱性初筛溶液: 称取供试品0.5 g,置具塞锥形瓶中,加碳酸钠试液(取无水碳酸钠10.5 g,加水溶解成100 mL)20.0 mL,振摇1 min,再加甲醇-水(250∶250, v/v)溶液230.0 mL,摇匀,超声10 min,冷却至室温,滤过,取滤液,即得。

UPLC-TOF-HRMS碱性推导溶液: 用甲醇-水(1∶1, v/v)溶液将碱性初筛溶液稀释制成每1 mL约含恩诺沙星500 ng的溶液,即得。

1.3.3 对照品溶液制备

UPLC-PDA对照品确证溶液: 取甲萘醌及亚硫酸氢钠甲萘醌对照品各约10 mg,精密称定,置100 mL量瓶中,加甲醇-水(1∶1, v/v)溶液溶解并稀释至刻度,摇匀;精密量取3 mL,置10 mL量瓶中,加甲醇-水(1∶1, v/v)溶液稀释至刻度,摇匀,即得。

UPLC-TOF-HRMS对照品确证溶液: 用甲醇-水(1∶1, v/v)溶液将UPLC-PDA对照品确证溶液稀释制成每1 mL含100 ng的溶液,即得。

## 2 结果与讨论

### 2.1 未知物的发现和初步分析

取UPLC-PDA酸性初筛溶液、碱性初筛溶液,按1.2.1节条件分别进样,见图1。酸性初筛溶液色谱图中除在2.376 min出现恩诺沙星主峰外,分别在1.870 min(未知物1)及5.122 min(未知物2)处出现未知色谱峰,最大吸收波长分别为229.0 nm及249.1 nm,将未知峰光谱图与常见兽药及非法添加物光谱图库进行比对,未发现匹配的结果。碱性初筛溶液色谱图中除恩诺沙星主峰外,未出现保留时间为1.870 min的未知峰,在5.122 min处出现与酸性初筛溶液色谱图中未知物2保留时间及光谱图一致的色谱峰,且峰面积增大近10倍。提示未知物1与未知物2在酸性/碱性条件下可能发生转化。

**图 1:**
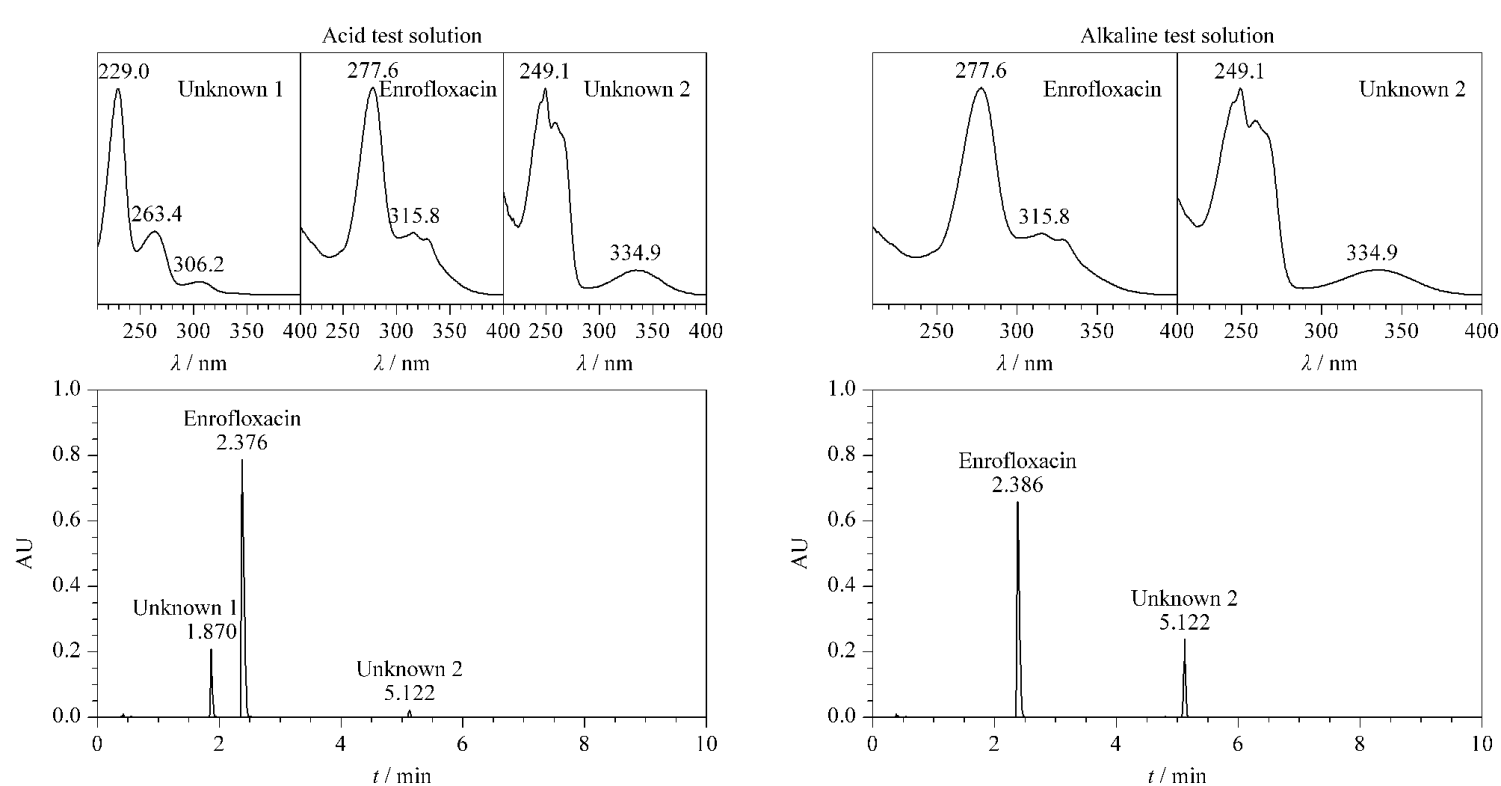
酸性初筛溶液和碱性初筛溶液的色谱图及紫外光谱图

### 2.2 未知物结构推断

按1.2.2节条件分别采集正离子模式和负离子模式下UPLC-TOF-HRMS碱性推导溶液、酸性推导溶液的色谱图,除恩诺沙星外,未检出与谱库相匹配的其他常见兽药或非法添加物。故借助SCIEX OS软件中的分子式拟合功能对未知物结构进行推导,并结合临床应用等其他信息作出推断。

2.2.1 未知物2结构推导

查看正离子模式下采集的碱性推导溶液数据,对总离子流图进行基峰提取(即把一段时间内响应比较高的信号提取出来,简称BPC),发现16.747 min处有与紫外色谱图中未知物2保留时间(*t*_R_=16.736 min)相近的一个峰(见图2),该峰的精确质荷比为173.0596(173.0598)(见图3)。

**图 2:**
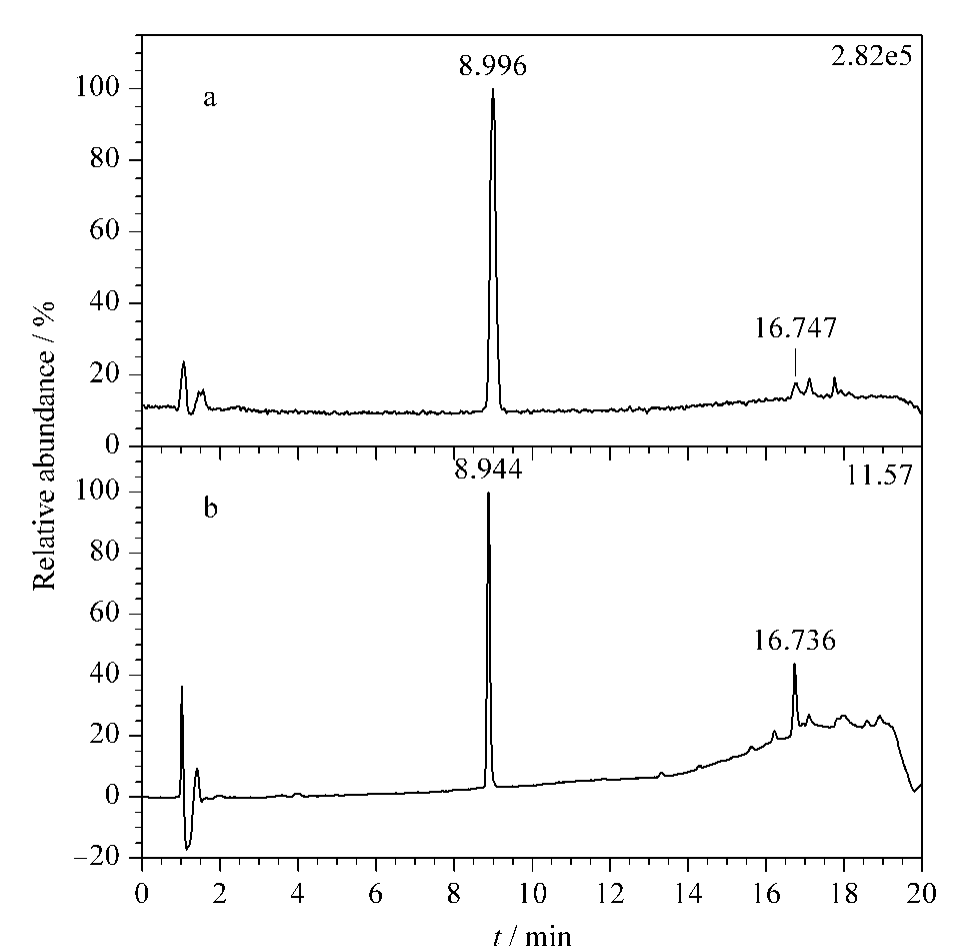
碱性推导溶液正离子模式下的(a)BPC图及(b)紫外色谱图

**图 3:**
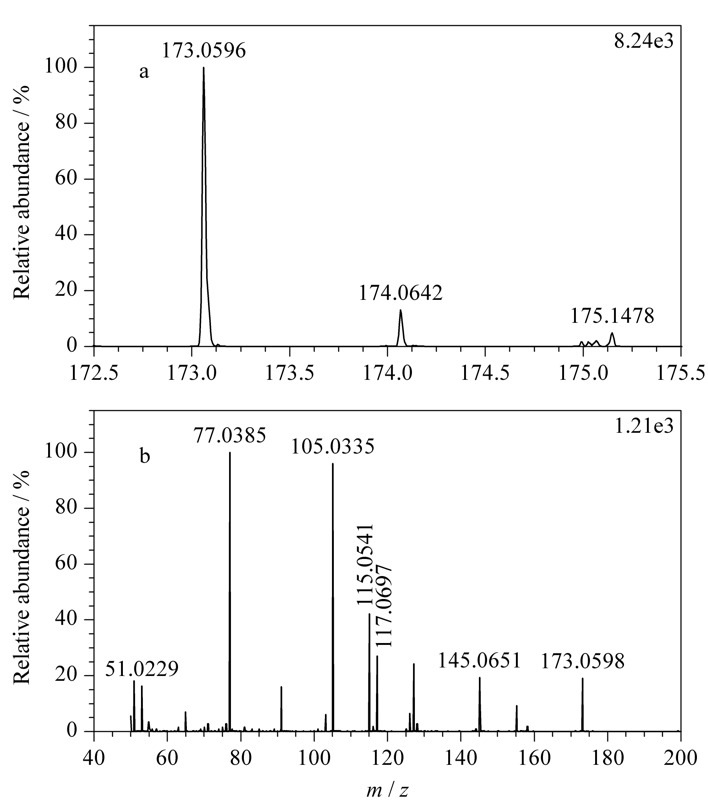
未知物2的(a)精确质荷比及同位素分布图和(b)二级碎片图

结合其同位素丰度比,元素组成设置为有机物常见的C、H、O、N、S、P、F,允许误差范围设置为5×10^-6^,软件计算给出唯一结果C_11_H_8_O_2_,质量相对误差-0.6×10^-6^,且同位素得分高,说明C_11_H_8_O_2_为未知物2分子式的可能性大。*m/z* 173.0598的母离子裂解产生*m/z* 145.0651、117.0697、115.0541、105.0335、77.0385及51.0229的二级特征碎片离子,见图3b;由特征碎片*m/z* 77.0385、51.0229,可知该化合物中含有苯环结构;特征碎片离子*m/z* 145.0651的解析为C_10_H_9_O^+^,由母离子*m/z* 173.0598中性丢失CO产生,母离子结构中含有羰基;特征碎片*m/z* 117.0697的解析为C_9_ $H_{9}^{+}$ ,比*m/z* 145.0651少CO,母离子结构中应含有两个羰基,并可能发生连续丢失CO的裂解行为;特征碎片*m/z* 115.0541的解析为C_9_ $H_{7}^{+}$ ,比*m/z* 145.0651少CH_2_O,推测母离子结构中连续丢失2个CO后发生重排;特征碎片*m/z* 105.0335的解析为C_7_H_5_O^+^,为苯环结构(-C_6_H_5_)加-CO,母离子结构中羰基应连接在苯环上。由上述二级特征碎片信息可知,母离子结构中含有一个苯环(-C_6_H_4_)及两个羰基(-CO),不饱和度为6,剩余部分不饱和度为2,推测剩余部分为丙烯基结构(-C_3_H_4_),且与其他结构成环。综上所述,母离子结构可能为图4a、b两种。由于结构a中两个羰基均与丙烯双键共轭,比结构b更稳定合理,推测未知物2为甲萘醌(见图4a)。

**图 4:**
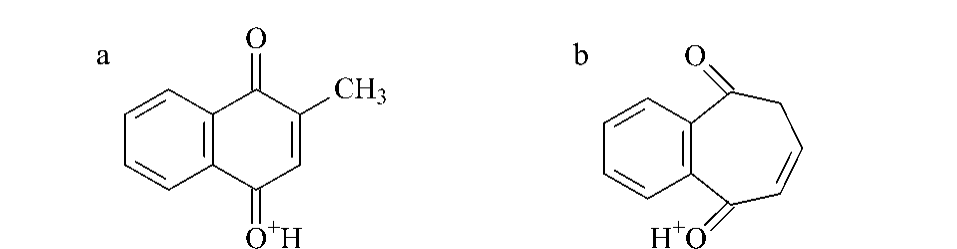
未知物2结构推测

2.2.2 未知物1结构推导

查看负离子模式下采集的酸性推导溶液数据,除恩诺沙星峰外,在6.940 min处可见一明显色谱峰(见图5a),与紫外色谱图中未知物1的保留时间(*t*_R_=6.862 min)相近(见图5b),该峰的精确质荷比为253.0174,结合其同位素信息(见图6a),得到该分子离子峰为C_11_H_9_O_5_S^-^;二级碎片谱图采集到*m/z*为80.9654的碎片(见图6b),软件给出的碎片离子为 ${\mathrm{HSO}}_{3}^{-}$ ,即亚硫酸氢根,中性丢失部分分子式为C_11_H_8_O_2_,与未知物2的推测分子式(甲萘醌)一致。结合甲萘醌3种常见的衍生物类型^[11]^(亚硫酸氢钠甲萘醌、亚硫酸氢烟酰胺甲萘醌和二甲基嘧啶醇亚硫酸甲萘醌),推测未知物1为亚硫酸氢钠甲萘醌。

**图 5:**
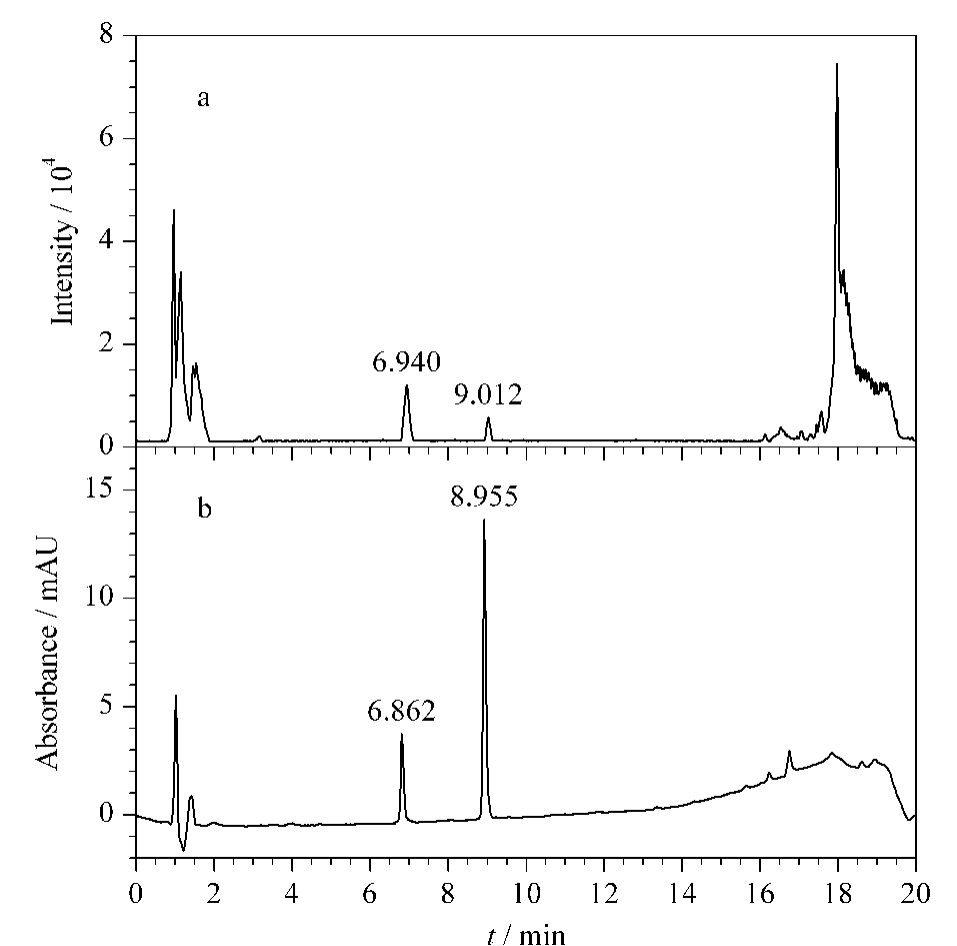
酸性推导溶液负离子模式下的(a)BPC图及(b)紫外色谱图

**图 6:**
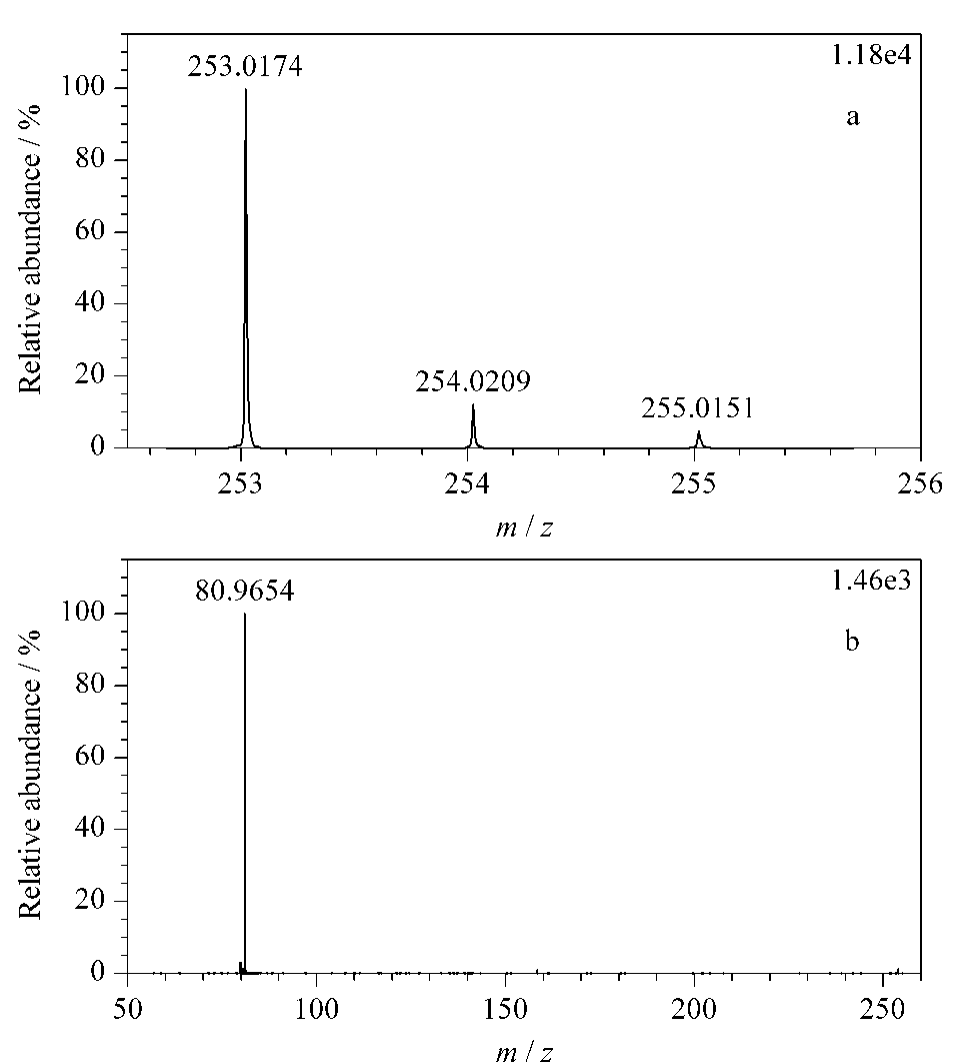
未知物1的(a)精确质荷比及同位素分布图和(b)二级碎片图

2.2.3 未知物的推断

从样品的临床应用及商品名入手,恩诺沙星粉(水产用)主要用于水产养殖动物的出血性败血症等^[12]^,在中国兽药信息网国家基础兽药库中查询该产品的商品名,多与止血作用相关,如“出血停”、“鱼血止”等^[13]^,与亚硫酸氢钠甲萘醌临床作用^[14]^相近。另有报道,亚硫酸氢钠甲萘醌在碱性溶液中析出甲萘醌^[15]^,为UPLC-PDA初筛的实验结论提供佐证。最后结合质谱分析结果,推测该未知物1为亚硫酸氢钠甲萘醌,未知物2为甲萘醌。为进一步增强质谱信息的可信度,推导了可能裂解的途径,见图7和图8。

**图 7:**
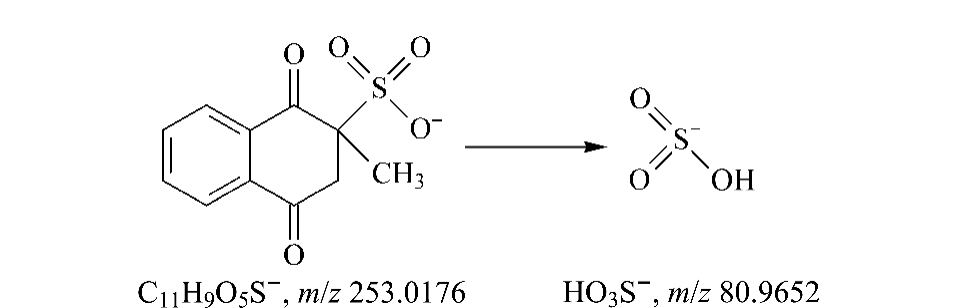
未知物1的裂解途径

**图 8:**
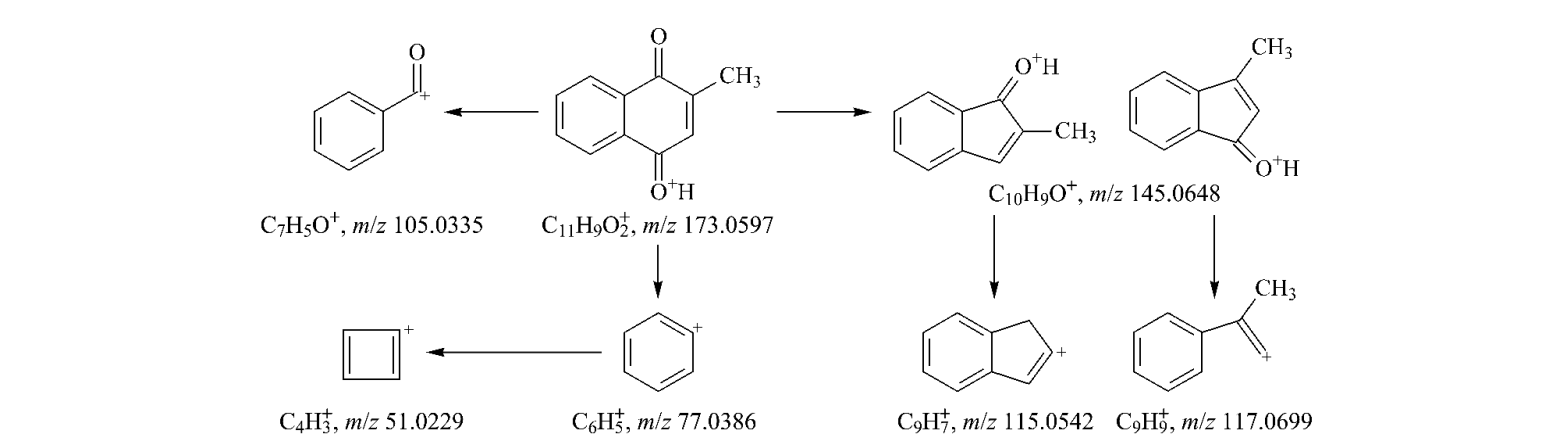
未知物2的裂解途径

### 2.3 未知物确证

2.3.1 HPLC-PDA法确证

将初筛溶液色谱图中的未知物1、未知物2色谱峰分别与UPLC-PDA对照品确证溶液中主峰的保留时间及紫外光谱进行比对(见图1和图9)。在该试验条件下,未知物1与对照品溶液中亚硫酸氢钠甲萘醌的保留时间一致,紫外光谱相似;未知物2与对照品溶液中甲萘醌的保留时间一致,紫外光谱相似。

**图 9:**
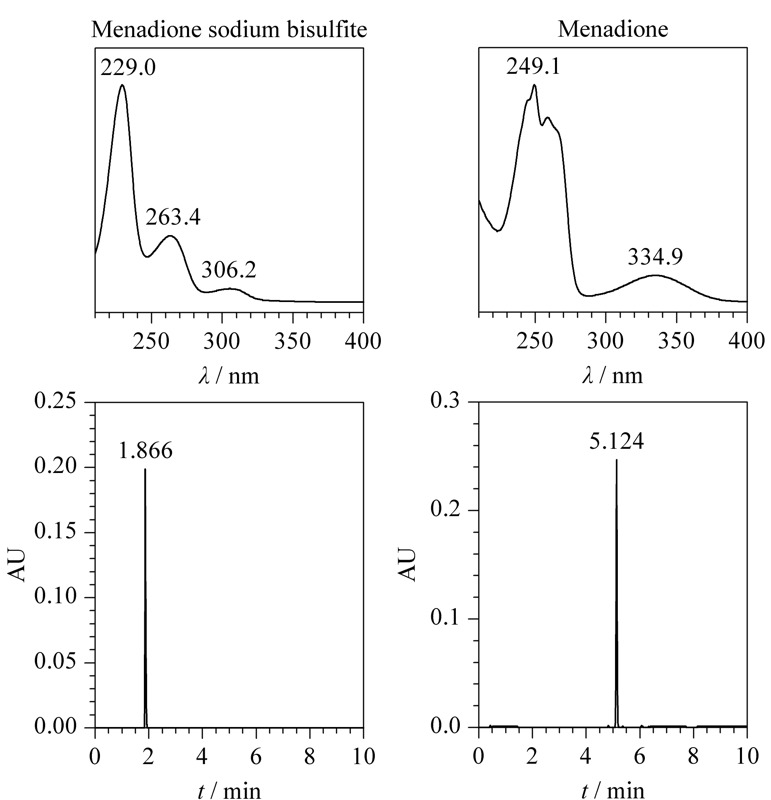
对照品溶液中亚硫酸氢钠甲萘醌和甲萘醌的色谱图及紫外光谱图

利用不同浓度本底添加对供试品中的未知物进行验证(取酸性初筛溶液与UPLC-PDA对照品确证溶液按1∶4、1∶1、4∶1的比例混合),使用Waters Empower 3软件进行峰纯度及库匹配检查,结果显示:添加前后,供试品色谱图各相应位置上的色谱峰的纯度角度均小于纯度阈值,可认为是单一物质峰;光谱相似度检查结果显示:添加前后,供试品色谱图相应位置上的色谱峰的光谱匹配角度均小于匹配阈值,表明与对照品的紫外光谱相似,可认为是同一化合物。判定未知峰1为亚硫酸氢钠甲萘醌,未知峰2为甲萘醌。峰纯度检查和光谱相似度检查结果见表1。

**表 1:** 未知物1峰纯度检查与光谱相似度检查结果

Analyte	Additive proportion	Purity angle	Purity threshold	Results of peak purity test	PDA matching angle	PDA matching threshold	Results of spectra similarity test
Acid test solution	-	0.225	0.282	All of purity angles are less than the threshold, which prove it is a peak of pure compound.	0.435	1.158	All of the PDA matching angles are less than the threshold, which prove its spectra is similar to the standard’s.
Acid test solution-standard solution of menadione sodium bisulfite	1∶4	0.084	0.287		0.091	1.158	
	1∶1	0.108	0.284		0.221	1.158	
	4∶1	0.228	0.297		0.328	1.158	

2.3.2 UPLC-TOF-HRMS法确证

在相同条件下,采集对照品母离子的精确质荷比、天然同位素分布、二级谱图、保留时间四大信息,载入软件谱库中。使用SCIEX MasterView软件将酸性推导溶液中提取到的未知物1信息与亚硫酸氢钠甲萘醌对照谱图进行比较,两者保留时间一致,一级质谱质量偏差为1.0×10^-6^,二级谱库匹配度为100%,见图10。将碱性推导溶液中提取到的未知物2信息与甲萘醌对照谱图进行比较,两者保留时间一致,一级质谱质量偏差为0.6×10^-6^,二级谱库匹配度为99.7%,见图11。按照农业农村部公告第289号《兽药中非特定非法添加物质检查方法》液相色谱-高分辨质谱法^[5]^进行结果判定,供试品色谱图中如出现与对照品峰保留时间一致的色谱峰(保留时间相对偏差不大于2.5%);供试品与对照品分子离子峰的质量数偏差不大于5×10^-6^,且二级质谱图与对照品的二级质谱图一致,判定该供试品中检出的未知物1为亚硫酸氢钠甲萘醌,未知物2为甲萘醌。

**图 10:**
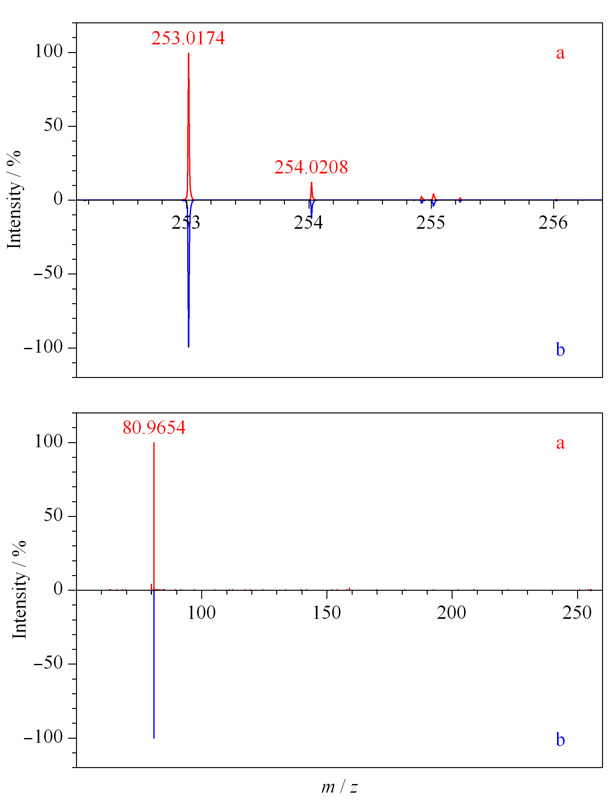
(a)未知物1与(b)谱库中亚硫酸氢钠甲萘醌对照品的匹配图

**图 11:**
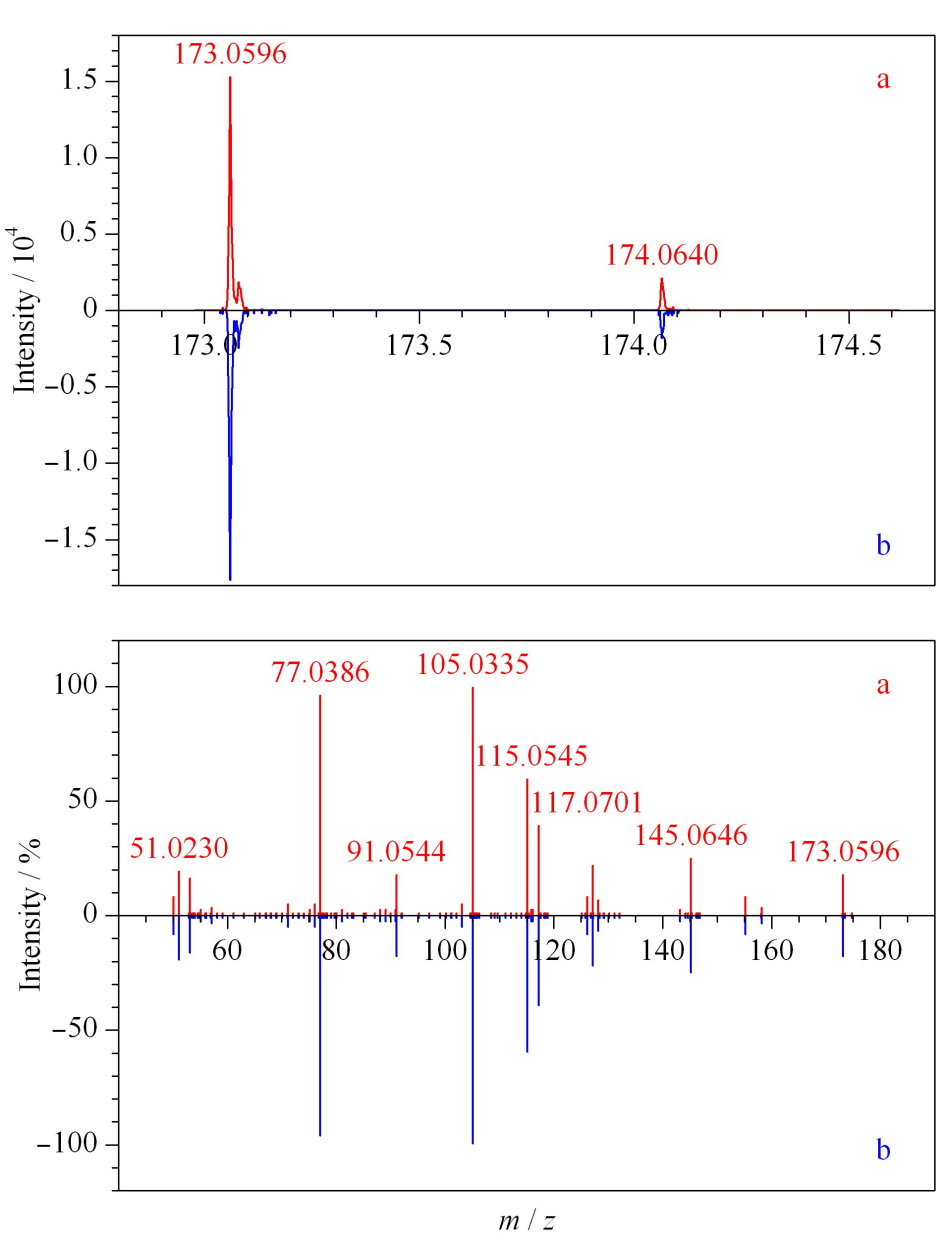
(a)未知物2与(b)谱库中甲萘醌对照品的匹配图

### 2.4 本研究中未知物推导确证思路

该样品中未知物1亚硫酸氢钠甲萘醌本身在正离子模式下没有响应,负离子模式下裂解产物仅为一个大的碎片离子,信息量过少,不易于进行化合物结构的推导。但其在碱性条件下可转换为甲萘醌,甲萘醌在负离子模式下无响应,但在正离子模式下有响应且碎片信息较多,可用于结构推导和解析,为未知物的确证提供了有力的佐证。

### 2.5 非法添加原因分析

恩诺沙星粉(水产用)临床上用于治疗水产养殖动物由细菌性感染引起的出血性败血症、烂鳃病、打印病、肠炎病、赤鳍病、爱德华氏菌病等疾病^[12]^。亚硫酸氢钠甲萘醌是水溶性维生素类药物,缺乏会影响凝血过程而引起出血,口服可直接吸收,达到止血作用,临床上常用于辅助治疗鱼、鳗等水产养殖动物的出血、败血症^[12]^。不法企业在恩诺沙星粉(水产用)中添加亚硫酸氢钠甲萘醌“增强”产品疗效,以期达到“多病同治”的效果。由于亚硫酸氢钠甲萘醌不稳定^[16]^,在存放过程中分解出甲萘醌,导致供试品中有少量甲萘醌检出。有文献报道在鱼类发生严重贫血、肝功能丧失的情况下,使用亚硫酸氢钠甲萘醌不但没有治疗作用,反而不利于病鱼的康复,甚至在治疗期间,使病情恶化,死鱼数量成倍增加^[17]^。故在恩诺沙星粉中非法添加亚硫酸氢钠甲萘醌可能引起潜在的、不可估量的危害。

## 3 结论

本文使用UPLC-TOF-HRMS及其数据处理软件,依靠其高质量精度和分子式预测功能,综合二级质谱解析和临床应用等信息,对一批兽药恩诺沙星粉(水产用)中的未知物进行化学结构鉴定,做出了合理推断。近年来,兽药中非法添加药物屡禁不止,相较于畜禽药,水产药因为关注度较低,已成为重灾区。本研究中样品按其质量标准正常检验,所有项目均符合规定,提示检验员对检验中出现的异常色谱峰应特别关注。HRMS能进行全质量数据采集,不仅能够一次性分析大量的目标化合物,还能在不用相关对照品的前提下检测多种非目标化合物^[18,19]^。通过HRMS技术进行定性筛查已成为一种趋势,现已有农业农村部公告第197号(可用于同时鉴别和确认150种兽药及其他化合物)^[20]^、《食品补充检验方法工作规定》BJS 201805(可同时筛查和定性确证90种那非类物质)^[21]^等多部利用高分辨质谱进行化合物筛查及确证的相关国家标准或行业标准出台,其强大的筛查功能将对不法分子产生极大的震慑作用,为行政执法提供充分的技术保障。
